# Prognostic impact of oral microbiome on survival of malignancies: a systematic review and meta-analysis

**DOI:** 10.1186/s13643-023-02419-7

**Published:** 2024-01-25

**Authors:** Shuluan Li, Tianyu Wang, Ya Ren, Zhou Liu, Jidong Gao, Zhi Guo

**Affiliations:** 1 Department of Nutrition, National Cancer Center/National Clinical Research Center for Cancer/Cancer Hospital & Shenzhen Hospital, Chinese Academy of Medical Sciences and Peking Union Medical College, Shenzhen, China; 2 Department of Breast Surgery, National Cancer Center/National Clinical Research Center for Cancer/Cancer Hospital & Shenzhen Hospital, Chinese Academy of Medical Sciences and Peking Union Medical College, Shenzhen, People’s Republic of China; 3 Department of Radiology, National Cancer Center/National Clinical Research Center for Cancer/Cancer Hospital & Shenzhen Hospital, Chinese Academy of Medical Sciences and Peking Union Medical College, Shenzhen, People’s Republic of China; 4 Department of Hematology, Huazhong University of Science and Technology Union Shenzhen Hospital, Guangdong, 518000 People’s Republic of China; 5 Institute of Infection, Immunology and Tumor Microenvironent, Hubei Province Key Laboratory of Occupational Hazard Identification and Control, Medical School, Wuhan University of Science and Technology, Wuhan, People’s Republic of China

**Keywords:** Oral microbiome, Malignancies, Survival, Meta-analysis

## Abstract

**Background:**

Recent studies have shown that there exists a significant correlation between oral microbiome and the occurrence of malignancies. However, the prognostic significance of oral microbiome for cancer patients remains unclear. The purpose of this meta-analysis is to evaluate the impact of oral microbiome on the survival of patients with malignant neoplasms.

**Methods:**

We conducted a thorough literature search of PubMed, Embase, and Cochrane Library databases until September 2022. The hazard ratio (HR) with a corresponding 95% confidence interval (CI) was analyzed using Review Manager 5.4 software for survival outcomes, including overall survival (OS), disease-specific survival (DSS), progression-free survival (PFS), and disease-free survival (DFS).

**Results:**

A total of 15 studies, covering 5191 samples with various types of cancers, were selected based on specified inclusion and exclusion criteria. In both univariate and multivariate analysis, patients with low diversity of the oral microbiome, or those with Fusobacterium-high/positive, or P. gingivalis positive in cancer tissue displayed poorer OS (univariate HR = 1.74; 95% CI 1.15–2.62; *P* = 0.009; multivariate HR = 1.56; 95% CI 1.07–2.27; *P* = 0.02), DSS (univariate HR = 2.06; 95% CI 1.50–2.84; *P* < 0.00001; multivariate HR = 1.80; 95% CI 1.48–2.20; *P* < 0.00001), and PFS/DFS (univariate HR = 2.00; 95% CI 1.12–3.58; *P* = 0.002; multivariate HR = 1.78; 95% CI 1.05–3.02; *P* = 0.003). Subgroup analysis revealed that Fusobacterium positive or high abundance in cancer tissues was associated with poor OS in multivariate analysis but had no statistical differences in PFS or DFS in univariate and multivariate analysis. Additionally, P. gingivalis positive in cancer tissue was also associated with worse OS.

**Conclusions:**

Our meta-analysis suggests that the composition of the oral microbiome may play a significant role in predicting survival outcomes for cancer patients.

**Supplementary Information:**

The online version contains supplementary material available at 10.1186/s13643-023-02419-7.

## Introduction

Cancer is the second leading cause of mortality worldwide, with nearly 10 million deaths recorded in 2020 [1]. Despite improved prognosis by the significant advancements in early detection and effective treatment, the 5-year survival rate remains at 40.5% [2]. How to accurately assess prognosis at diagnosis remains challenging, hindering the tailoring of treatment plans and monitoring for recurrence. To address this issue, further studies are needed to explore new biomarkers that can predict cancer prognosis.

Recent research has highlighted the role of oral microbiota, the most diverse microbiota in the human body in the development of various malignancies [3]. Evidence suggests that some oral taxa, including Fusobacterium nucleatum and Porphyromonas gingivalis may promote carcinogenesis through mechanisms, such as inflammation induction, immunosuppression, promotion of malignant transformation, antiapoptotic activity, and secretion of carcinogens [4–6]. Furthermore, several studies have demonstrated the association between certain oral microbiota and cancer prognosis [7]. For example, Fusobacterium species have been shown to be positively linked to poor prognosis in pancreatic cancer, oral squamous cell carcinoma, and colorectal cancer [8–10]. Mohamed et al. found that higher salivary carriage of the genus Candida was associated with poor prognosis, while Malassezia was enriched in patients with favorable prognosis in oral squamous cell carcinoma [11]. Du et al. demonstrated that lower within-community diversity of the oral microbiome was associated with higher mortality in nasopharyngeal carcinoma prognosis, especially in elderly cases [12]. Wei et al. suggested that Fusobacterium nucleatum and Bacteroides fragilis were more abundant in worse prognosis groups [13]. However, due to differences in study design, sample size, study population, and research region, the impact of oral microbiota on cancer prognosis remains unclear. To address this gap, we conducted a meta-analysis encompassing various types of cancer, to provide comprehensive evidence and clarify the association between oral microbiota and cancer prognosis. This study aims to enhance understanding of the impact of oral microbes on the survival of cancer patients and provide avenues for improving patient outcomes.

## Methods

This meta-analysis adhered to the guidelines outlined in the Preferred Reporting Items for Systematic Reviews and Meta-Analyses (PRISMA) [14].

### Search strategy

The study conducted a comprehensive search of the relevant literature using PubMed, Embase, and Cochrane Library databases until September 2022. Supplementary Material provides an overview of the search terms employed. Additionally, the references of relevant studies were screened to identify additional studies. Two authors (SL and YR) independently searched and scrutinized all potentially relevant studies from each eligible report. Any discrepancies were resolved via consensus between the two authors.

### Inclusion criteria

The following criteria were employed to select studies that were eligible for inclusion: (1) patients: studies involving patients diagnosed with malignant neoplasms via pathological examination. Microbiota that originated from the oral cavity, or bloodstream, or distant tissues that spread from the oral cavity were assessed. (2) Intervention methods: studies that conducted the detection of oral microbiomes using 16S rRNA sequencing, polymerase chain reaction (PCR), or other techniques. Samples included saliva, serum, cancer tissue, and normal tissue. Patients received chemotherapy, radiation therapy, or surgical treatment. (3) Comparison factor: studies that investigated the correlation between the existence, abundance, diversity, or components of microbiota and the cancer survival rate. (4) Outcome measures: studies that reported the clinical outcomes of patients in terms of overall survival (OS), disease-specific survival (DSS), progression-free survival (PFS), and disease-free survival (DFS) using hazard ratio (HR) and 95% confidence interval (CIs). (5) Study design: only randomized controlled clinical trials and observational research were included. (6) Full-text availability was mandatory.

### Exclusion criteria

The exclusion criteria utilized in this study were: (1) patients diagnosed with benign neoplasms. (2) microbiota did not originate from oral cavities, such as gastrointestinal microbes or HPV. (3) unavailable data on OS, DSS, PFS/DFS. (4) absence of a control group. (5) abstracts, letters, comments, reviews, case reports, meta-analyses, or nonclinical studies, and (6) unavailability of the full text was unavailable.

### Data extraction and quality assessment

Two reviewers (SL and YR) independently recorded information such as first author name, publication country, publication year, sample size, patient age, tumor stage, treatment, microbiota evaluation method and comparison factors, OS, DSS, PFS, and DFS and their corresponding HRs and 95% CIs. Discrepancies were resolved through discussion consensus was reached by three authors (SL, TW, and YR) on study exclusion. The quality of eligible studies was assessed based on the Newcastle–Ottawa Scale (NOS) criteria. Any discrepancies in data extraction and quality assessment were resolved through consensus.

### Statistical analysis

The association of oral microbiomes with cancer prognosis was evaluated by pooling HRs and their respective 95% CIs. Based on the reported HR values and 95% confidence interval (95% CI) in the studies included, lnHR and its standard error were calculated according to the method by Parmar et al. [15], and then the HRs were pooled. Heterogeneity across studies was assessed using the Cochran *Q* test and *I*^2^ statistics. When *I*^2^ < 25%, no heterogeneity; *I*^2^ ≥ 25% and < 50%, mild heterogeneity; *I*^2^ ≥ 50% and < 75%, moderate heterogeneity; *I*^2^ ≥ 75%, significant heterogeneity. If heterogeneity was significant, a fixed-effect model was used. Sensitivity analysis was performed removing a single study in each turn to evaluate the impact of individual studies on the pooled estimates. Publication bias was assessed by Begg’s funnel plot and if *P* > 0.05 was considered to be lack of publication bias. Statistical analyses were employed using the Review Manager 5.4 software. All statistical tests were two-tailed, and significance was set at *P* < 0.05.

## Results

### Study selection and characteristics

**Fig. 1:**
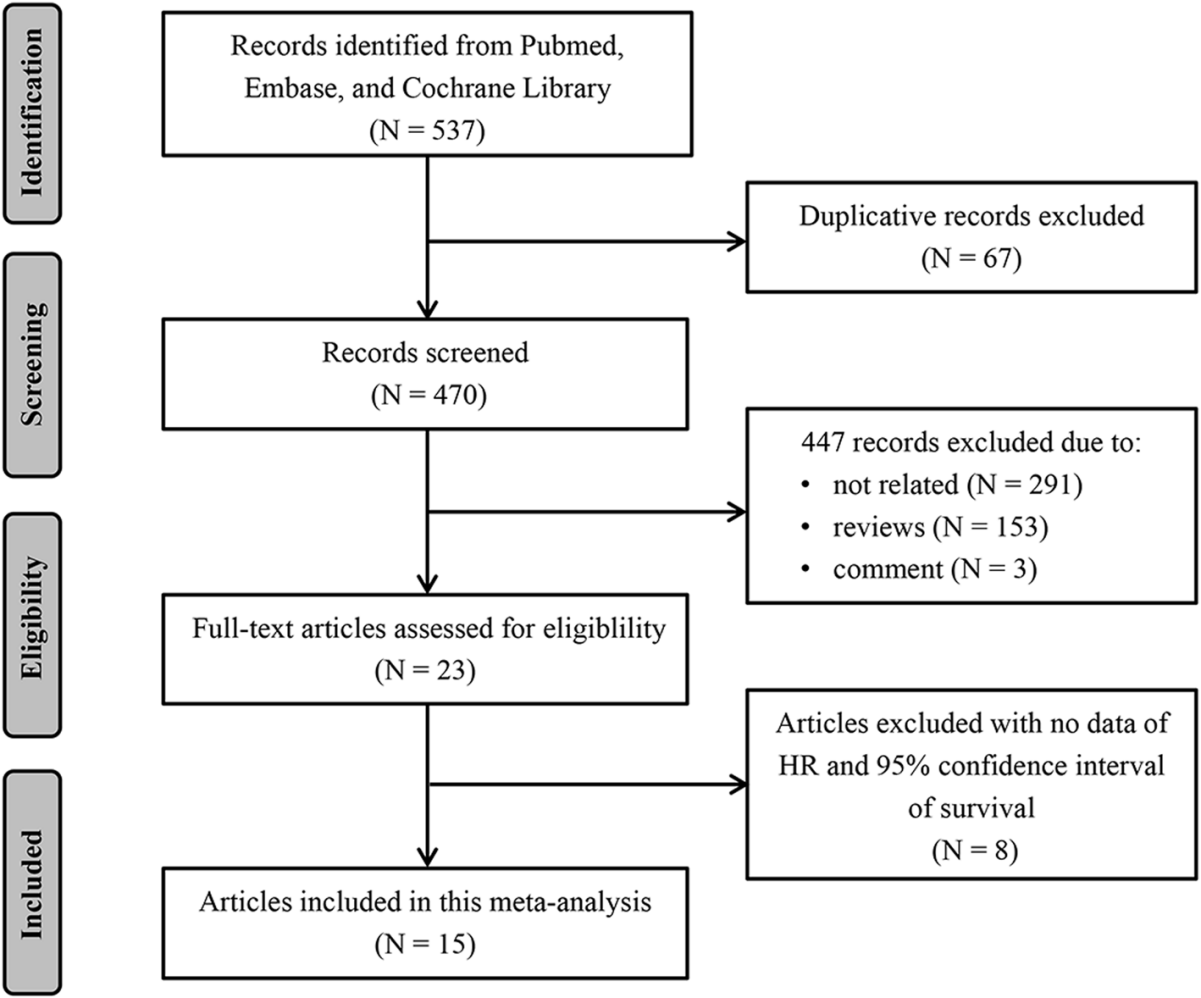
A flow chart of the studies selection process

Initially, we conducted a comprehensive search for relevant studies in four electronic databases, namely, PubMed, Embase, Web of Science, and the Cochrane Library, resulting in the identification of 537 articles. Subsequently, through a screening of titles and abstracts, we excluded 67 articles due to repetition, and 291 articles because they were irrelevant studies. After a thorough review of the full texts of the remaining 179 articles, 153 reviews, 3 comments, and 8 articles without data on HR values concerning prognosis were excluded, Eventually, 15 eligible studies were included in the meta-analysis. The detailed search and study selection process is shown in Fig. 1. Of the 15 studies selected, 8 studies had a prospective cohort design and 7 had a retrospective cohort design. Encompassing a total of 5191 samples across 15 studies with cancers such as nasopharyngeal carcinoma, oral squamous cell carcinoma, pancreatic cancer, esophageal cancer, and colorectal cancer included. Out of these studies, 10, 5, 5, and 2 studies evaluated OS, DSS, DFS, and PFS, respectively. Table 1 presents the summaries of the main characteristics of the 15 included studies [8–13, 16–24].

**Table 1:** Characteristics of included studies

Author/ year	Country	Sample size cases/controls	Samples	Type of cancer	Detection Method	Microbiome	Outcome	Multivariate analyses	Study design	NOS score
Mima/2016 [8]	USA	1069	tissue	CRC	PCR	F. nucleatum	OS, DSS	The amount of Fusobacterium nucleatum DNA	P	7
Neuzillet/2021 [9]	France	151	tissue	OSCC	16S rRNA sequencing	F. nucleatum	PFS, OS	F. nucleatum status, UICC stage, TP53 mutational status	R	6
Mitsuhashi/2015 [10]	Japan	283	tissue	PC	TaqMan gene expression assay	F. nucleatum	DSS	Fusobacterium species, CIMP status, and expression level of microRNA-31	P	6
Mohamed/2021 [11]	Norway	72 (59/13)	saliva	OSCC	NGS	genus Malassezia	OS	salivary carriage of both Candida and Malassezia, age	P	7
Du/2022 [12]	Sweden	482	saliva	NPC	16S rRNA sequencing	microbial diversity	OS, DSS	Faith’s PD diversity, Observed ASVs diversity, Shannon diversity	P	7
Wei/2016 [13]	China	180	tissue	CRC	RNAsequencing and PCR	F. nucleatum	DFS, OS	CEA, TNM stage, B. fragilis, F. nucleatum	P	7
Lee/2018 [20]	Korea	246	tissues	CRC	PCR	F. nucleatum	DFS, OS	Differentiation, CIMP, Metastatic site, F. nucleatum amount	P	6
Jeong Oh/2018 [17]	Korea	593	tissues	CRC	PCR	F. nucleatum	DFS	No	R	7
Chen/2020 [18]	Taiwan	156	oralbiofilms	ESCC	PCR	P. gingivalis	OS	Clinical stage, P. gingivalis staining, Response to Neoadjuvant, Treatment	P	8
Yu/2018 [25]	China	196	tissue	CRC	RNA-sequencing and PCR	F. nucleatum	RFS	F. nucleatum abundance, AJCC stage	R	6
Qiao/2022 [24]	China	802	tissue	NPC	16S rRNA sequencing and PCR	bacterial load	DFS, OS	sex, age, stage, pathological type, intratumoral bacterial load, and chemotherapy	R	7
Gao/2016 [16]	China	130 (100/30)	tissue	ESCC	PCR	P. gingivalis	OS	No	P	7
Yamamura/2016 [17]	Japan	325	tissues	ESCC	PCR	F. nucleatum	DSS	Performance status, Tumor stage, Fusobacterium	R	5
Yan/2017 [18]	China	280	tissues	CRC	PCR	F. nucleatum	DFS, DSS	Tumor invasion, Lymph node metastasis, Distant metastasis, Serum CEA level, Fusobacterium level	R	6
Gao/2018 [19]	China	226 (146/80)	serum	ESCC	ELISA	P. gingivalis	OS	N-stage, IgG (EU), IgA (EU)	P	7

*CRC* colorectal cancer, *DFS *disease free survival, *DSS *Disease Free Survival, *ELISA *enzyme-linked immunosorbent assay, *ESCC *esophageal cancer, *F. nucleatum *Fusobacterium nucleatum, *NGS *next generation sequencing, *NPC *nasopharyngeal carcinoma, *OS *overall survival, *OSCC *oral squamous cell carcinoma, *P* Prospective Cohort, *PC* pancreatic cancer, *PCR* polymerase chain reaction, *PFS *progression free survival, *P. gingivalis *Porphyromonas gingivalis, *R *Retrospective Cohort, *RFS *Recurrence free survival. *CIMP* CpG island methylator phenotype

### The impact of oral microbiomes on survival

The aim of our study was to perform a meta-analysis of the impact of oral microbiomes on the survival of cancer patients, including OS, DSS, DFS, and PFS. Our results indicate that patients with low diversity of oral microbiomes, or Fusobacterium positive or high abundance, or P. gingivalis positive in cancer tissues experienced poorer clinical outcomes compared to patients with high diversity of oral microbiomes, or those with negative/low levels of Fusobacterium, or negative level of P. gingivalis-negative.

### Overall survival (OS)

**Fig. 2:**
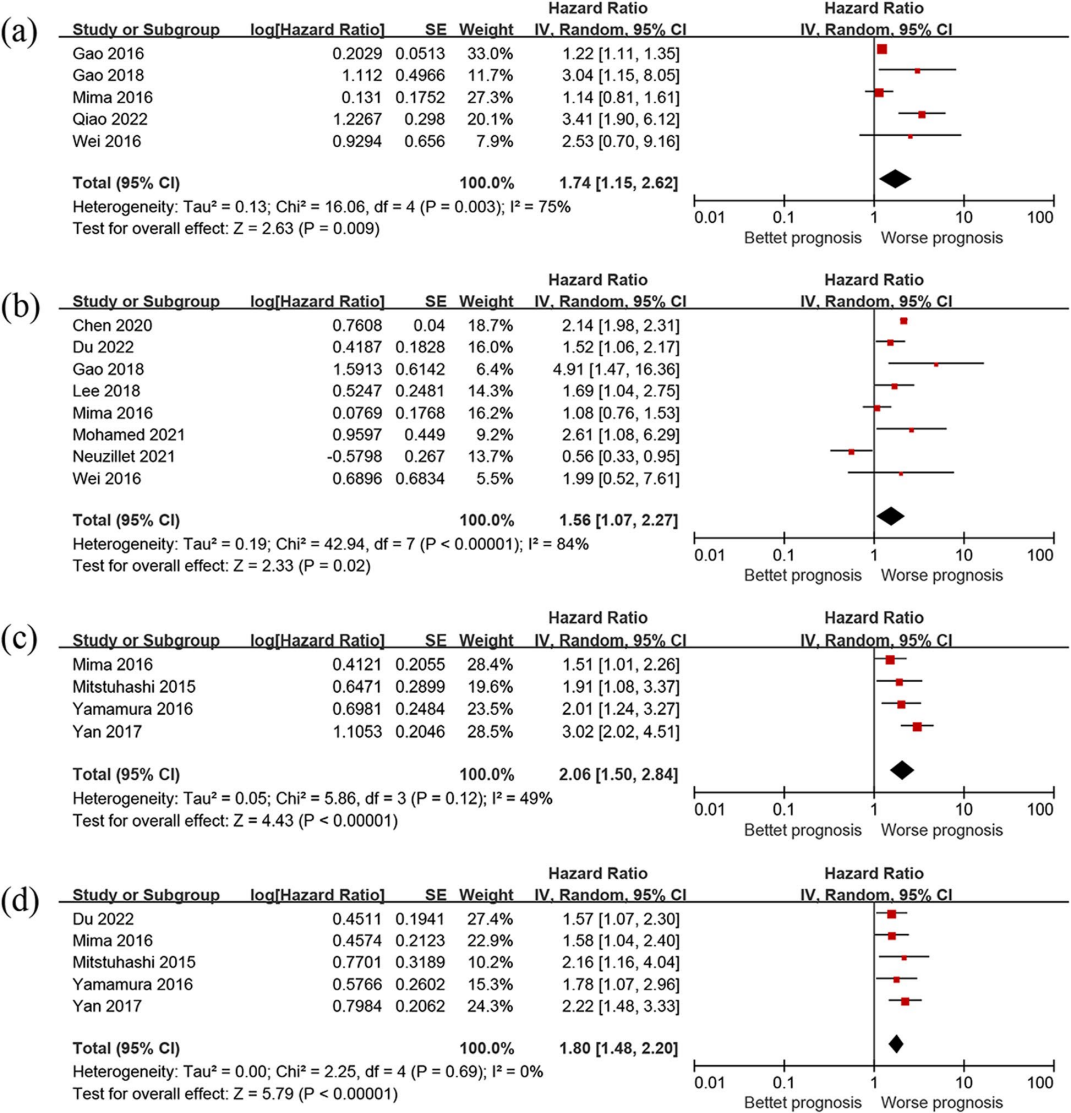
Forest plot of the impact of oral microbiome on clinical efficacy. **a** Oral microbiome and OS (by univariate HR). **b** Oral microbiome and OS (by multivariate HR). **c** Oral microbiome and DSS (by univariate HR). **d** Oral microbiome and DSS (by multivariate HR)

With regards to OS or DSS, studies that lacked HR data or reported incomplete survival curve data were excluded. As a result, 5 studies with a total of 2407 samples conducted univariate Cox proportional hazards regression analysis, and 8 studies with a total of 2582 samples conducted multivariate HR analysis. Our meta-analysis suggests a significant association between low diversity of oral microbiome, Fusobacterium positive or high abundance, or P. gingivalis positive in cancer tissues and poor OS (univariate HR = 1.74, 95% CI 1.15–2.62, *P* = 0.009; multivariate HR = 1.56, 95% CI 1.07–2.27, *P* = 0.02). Univariate analysis demonstrated moderate heterogeneity across the included studies (*P* = 0.003, *I*^2^ = 75%), whereas, multivariate analysis showed high heterogeneity (*P* < 0.00001, *I*^2^ = 84%) (Fig. 2). Due to the moderate and high heterogeneity of the results, a random-effect model was adopted, which produced similar results.

### Disease-specific survival (DSS)

Regarding DSS, we examined 4 studies encompassing a total of 1957 samples for univariate analysis and 5 studies comprising a total of 2439 samples for multivariate analysis of DSS. Our meta-analyses demonstrated that patients with a low diversity of oral microbiome, Fusobacterium positive or high abundance, or P. gingivalis positive in cancer tissues had poorer DSS (univariate HR = 2.06, 95% CI 1.50–2.84, *P* < 0.001, multivariate HR = 1.80, 95% CI 1.48–2.20, *P* < 0.001). The heterogeneity was low between studies in univariate analysis (*P* = 0.12, *I*^2^ = 49%), and non-existent in multivariate analysis (*P* = 0.69, *I*^2^ = 0%), (Fig. 2).

### Disease-free survival (DFS) and progression-free survival (PFS)

**Fig. 3:**
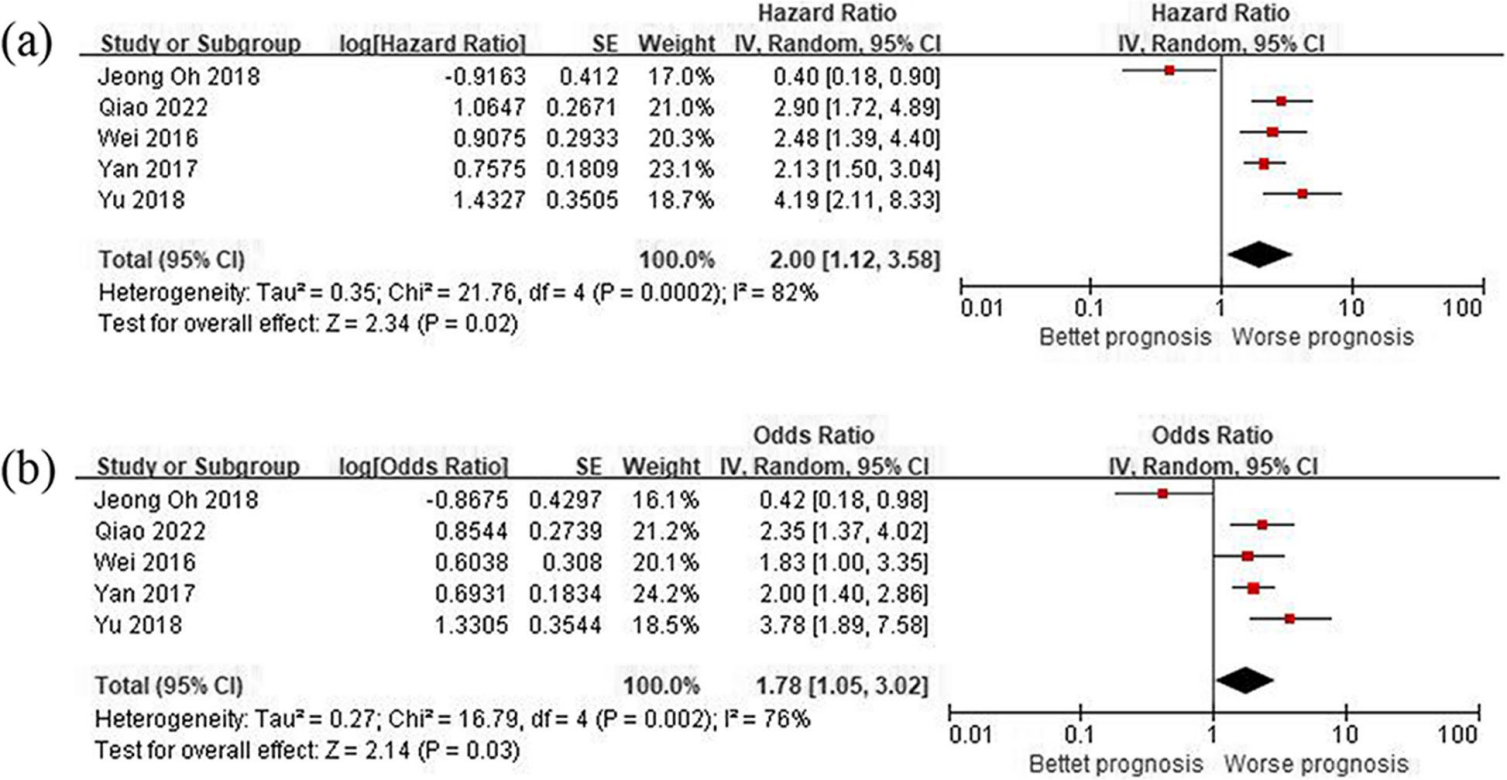
Forest plot of impact of oral microbiome on clinical efficacy. **a** Oral microbiome and DFS or PFS (by univariate HR). **b** Oral microbiome and DFS or PFS (by multivariate HR)

Owing to the limited number of articles that assessed DFS or PFS, a combined analysis of DFS and PFS was conducted. The findings indicated a significant association between a low diversity of oral microbiome, Fusobacterium positive or high abundance, or P. gingivalis positive in cancer tissues and worse DFS or PFS (univariate HR = 2.00, 95% CI 1.12–3.58, *P* = 0.02; multivariate HR = 1.78, 95% CI 1.05–3.02, *P* = 0.003). The presence of significant interstudy heterogeneity was observed in both univariate analysis (*P* = 0.0002, *I*^2^ = 82%) and multivariate analysis (*P* = 0.002, *I*^2^ = 76%) (Fig. 3). Similar results were obtained when a random-effect model was used.

### Subgroup analysis

**Fig. 4:**
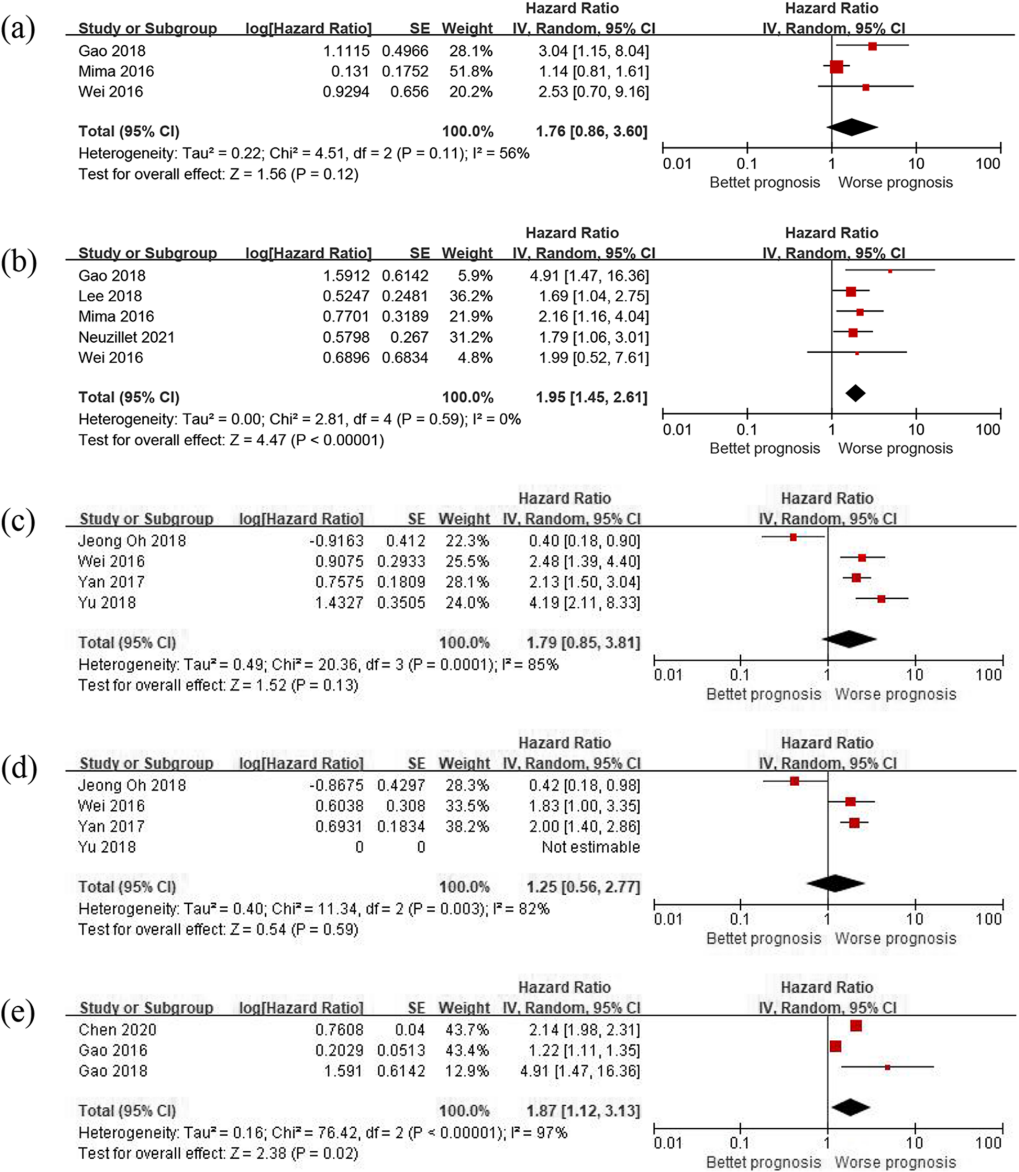
Subgroup analysis Fusobacterium on clinical efficacy. **a** Fusobacterium and OS (by univariate HR). **b** Fusobacterium and OS (by multivariate HR), **c** Fusobacterium and PFS or DFS (by univariate HR). **d** Fusobacterium and PFS or DFS (by multivariate HR). **e** Subgroup analysis P. gingivalis on OS

Furthermore, subgroup analysis was carried out for Fusobacterium subgroups and the effect of Fusobacterium on clinical outcomes was determined. The results showed that Fusobacterium positive or high abundance in cancer tissues had no statistically significant effect on OS in univariate analyses with an HR and 95% CI of 1.76 (0.86–3.60, *P* = 0.12) exhibiting moderate heterogeneity (*P* = 0.11, *I*^2^ = 56%), but was a poor prognostic factor for OS in multivariate analyses, with an HR and 95% CI of 1.95 (1.45–2.61, *P* < 0.001) with no heterogeneity (*p* = 0.59, *I*^2^ = 0%). Fusobacterium showed no effect on PFS or DFS in both univariate and multivariate analysis (*p* = 0.13 and 0.59) (Fig. 4).

The impact of P. gingivalis on OS was also studied. The result revealed that P. gingivalis positive in cancer tissue had a worse prognostic impact with an HR and 95% CI of 1.87 (1.12–3.13, *P* = 0.02). However, high heterogeneity was observed (*P* < 0.001, *I*^2^ = 97%) (Fig. 4).

**Fig. 5:**
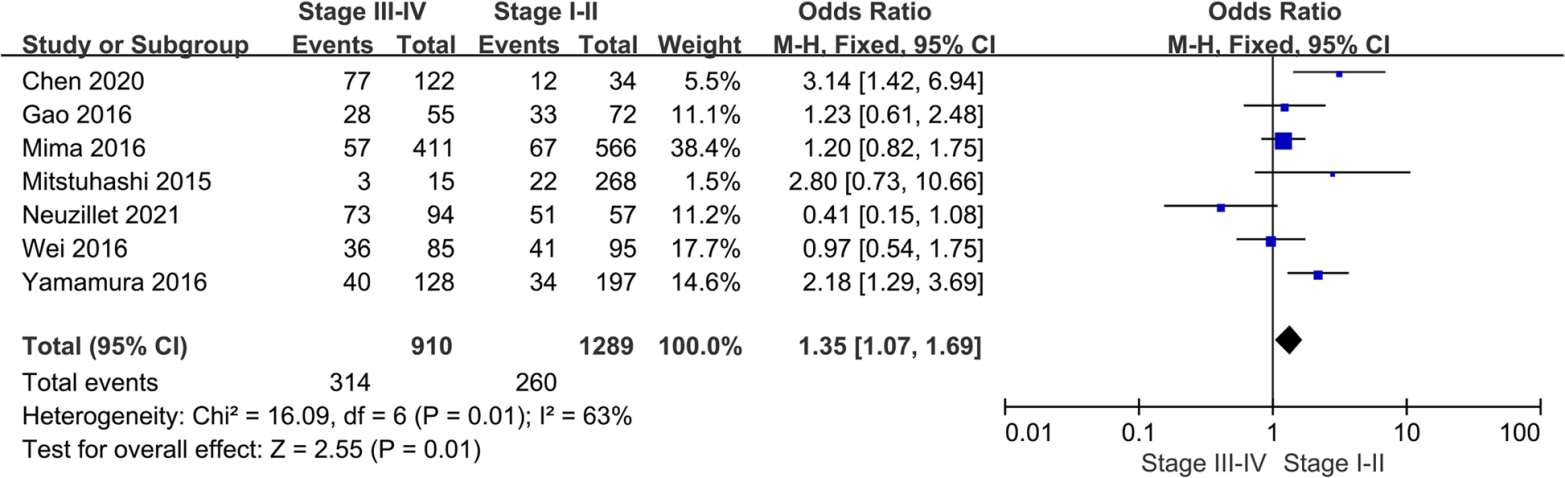
Funnel plot of relation between oral microbiome and tumor stage

The relation of tumor TNM staging and oral microbiome was also performed, the results showed that tumor staging was associated with oral microbial infection. The positive rate of patients in stage III–IV was 1.35 times higher than that in stage I-II, with an ORR and 95% CI of 1.35 (1.07–1.69, *p* = 0.01) (Fig. 5).

### Assessment of quality and risk of bias in included studies and publication bias

**Fig. 6:**
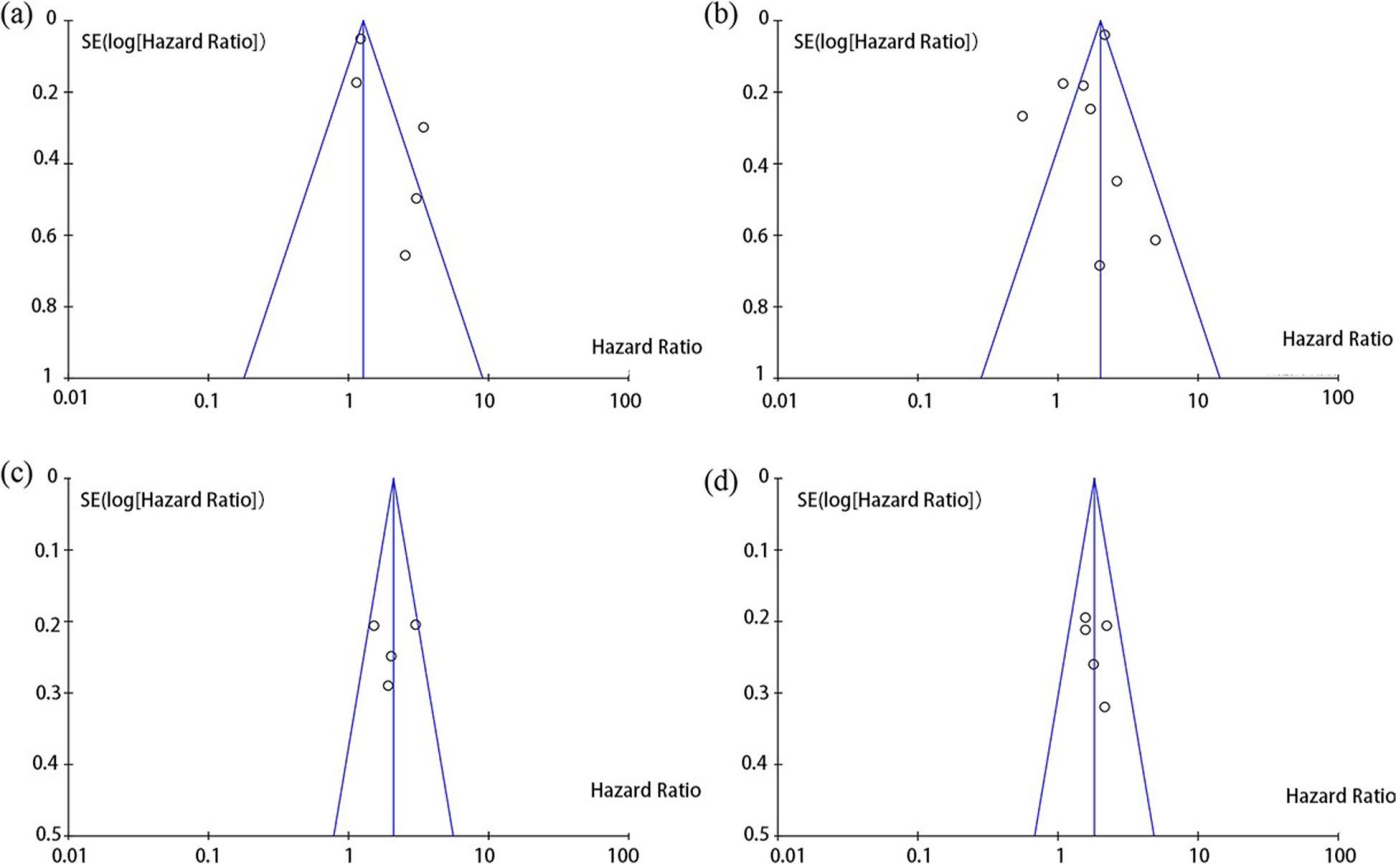
Funnel plot of the impact of publication bias. **a** Oral microbiome and OS (by univariate HR). **b** Oral microbiome and OS (by multivariate HR). **c** Oral microbiome and DSS (by univariate HR). **d** Oral microbiome and DSS (by multivariate HR )

The 9-point Newcastle–Ottawa (NOS) scale was utilized to undertake quality assessment and risk of bias analysis. Table 1 displays the evaluation outcomes. Begg’s funnel plot was employed to examine publication bias associated with survival, and Fig. 6 illustrates that no significant publication bias was found in these studies (Fig. 6).

## Discussion

This systematic review and meta-analysis included 15 global studies that encompassed 5191 samples exhibiting various types of cancer, which investigated the impact of the oral microbiome on survival, as measured by OS, DSS, DFS, and PFS. The findings demonstrate that cancer patients with low oral microbiome diversity, high/positive oral Fusobacterium abundance, or positive Porphyromonas gingivalis in cancer tissue had poorer OS. Subgroup analysis in Fusobacterium subgroups revealed that Fusobacterium positivity or high abundance in cancer tissues were poor prognostic factors for OS in multivariate analysis, but they had no effect on OS, PFS, or DFS in univariate analysis. The impact of P. gingivalis on OS was also examined and P. gingivalis positivity in cancer tissues was found to be associated with a poorer prognosis. Therefore, the diversity of oral microbiomes and specific flora are associated with tumor prognosis, and their simple detection and assessment make them promising biomarkers for evaluating tumor prognosis.

The oral microbiome is a highly complex ecosystem, encompassing a vast number of bacterial species. The oral cavity of an average adult, there are 5–10 billion bacteria, consisting of approximately 200 dominant species and 500 minor species [25]. In the past three decades, this intricate ecosystem has been studied extensively through the utilization of the Human Microbiome Project and high-throughput sequencing of genes, allowing for a comprehensive survey of the human oral microbiota. Pathogenic bacteria such as Streptococcus, Prevotella, Fusobacterium, Porphyromonas gingivalis, and Capnocytophagy have been identified to be commonly present. Furthermore. Studies have shown that the abundance, diversity, and structure of oral microorganisms in the saliva of tumor patients or tumor tissues can differ markedly [26]. Research has found that patients with esophageal squamous cell carcinoma have lower diversity of salivary flora, with Prevotella, Streptococcus, and Porphyromonas showing a relative abundance increase. Additionally, the detection rate of Porphyromonas gingivalis in esophageal squamous cell carcinoma lesions is as up to 61%, compared to a mere 12% in adjacent regions [21]. In a study comparing the oral flora of 361 pancreatic cancer patients and 371 non-pancreatic cancer patients, Fan et al. discovered that the detection rate of Porphyromonas gingivalis and Actinobacillus actinomycetes is higher in the oral cavity of patients with pancreatic cancer, with reduced abundance [27]. Moreover, studies have identified significant differences in the oral flora of liver cancer patients and healthy individuals, with liver cancer patients displaying a higher diversity of oral flora and different flora composition [28]. Multiple studies have demonstrated the involvement of the microbiota in the tumorigenesis and progression of various cancers through inflammation-mediated immunosuppression, metabolic pathways, and bacterial-derived toxins [3, 4, 7, 26]. Our analysis supports the notion that the oral microbiota is highly associated with the digestive tract, digestive gland, or related tumors, Specifically, our meta-analysis found that nasopharyngeal carcinoma, oral squamous cell carcinoma, esophageal carcinoma, pancreatic carcinoma, and colorectal cancer were all linked to the oral microbiota. These cancers are notorious for their high morbidity and high mortality rates, with most patients presenting at advanced stages at diagnosis that preclude surgical interventions. As conventional treatments (adjuvant treatments) have limited efficacy, identifying sensitive and easily detectable tumor markers is critical. In this regard, the oral microbiota-tumor interaction presents a promising avenue for developing new biomarkers and therapeutic targets across various types of cancer. Nonetheless, while many indicators are used to monitor tumor progression and prognosis, the value of oral microbiota in this context warrants further investigation.

This meta-analysis examined the correlation between the oral microbiota and cancer prognosis. revealing that the diversity of oral microorganisms and the abundance/positivity of certain microorganisms were associated with patient survival outcomes. Notably, Bingula et al. reported that patients with non-small cell lung cancer exhibited richer and more uniform oral microbiota diversity in normal tissue, which corresponded with a lower recurrence rate and higher disease-free survival rate [29]. Additionally, higher oral microbial alpha diversity in pancreatic tumor tissue from patients with pancreatic cancer was associated with longer overall survival [30]. Sims et al. found that the microbiome diversity index was an independent predictor of overall survival and recurrence-free survival after chemotherapy in cervical cancer cases [31]. The underlying reasons for these results include the important role of oral microbiome diversity and balance in the immune function of the oral mucosa in protecting the host from foreign attack and disease development [32]. Previous studies on the dynamics of oral bacterial communities by Yang et al. showed that the abundance and function of oral bacterial communities increased with increasing stages of oral squamous cell carcinoma [33]. There are 10 studies including tumor stage, 4 CRC studies, 4 ESCC studies, 1 OSCC study, and 1PC study in our analysis. Of which 6 studies had a statistically significant effect, 4 studies had no statistically significant effect. Among them, three studies only had T and N staging, without TNM staging. Then, we analyzed meta-analyses of the remaining seven studies containing TNM staging (including 2 CRC, 3 ESCC, 1 PC, and 1 OSCC study) and found that tumor staging was associated with oral microbial infection. The positive rate of patients in stage III-IV was 1.35 times higher than that in stage I–II. Oral microbes modulate innate immune signaling and specific microRNAs, activate autophagy pathways, and alter chemotherapy response. Applied bioinformatics and functional studies by TaChung Yu confirmed that Clostridium nucleatum promotes drug resistance in colorectal cancer to chemotherapy [19]. The oral microbiome may also be responsible for the occurrence of adjuvant therapy complications such as oral mucositis and gastrointestinal symptoms [34–36], which may lead to discontinuation of adjuvant therapy such as chemotherapy or radiotherapy and malnutrition, ultimately resulting in a poor prognosis for patients [37].

The oral microbiota has been detected in various sites of the body, such as the oral cavity, tumor surface, and intratumoral tissue. It is possible that the oral microbiota is transmitted through a specific route. Furthermore, the oral microbiota plays a significant role in tumor formation and composition that changes during tumor progression. Saliva testing for the oral microbiota is easy. a non-invasive, cost-effective diagnostic tool that shows great promise in the development of biomarkers monitoring health and disease and personalized medicine. Investigating the interaction mechanism between the oral microbiota and tumor development can provide important guidance for the early detection of tumors and long-term survival outcomes Additionally, the oral microbiota acts as the first barrier for the human body, and exploring the role of different microorganisms in mediating immune responses holds great potential. Therefore, further research on the oral microbiota and tumor prognosis can be of great benefit to cancer patients.

Our study conducted a strict analysis of the included studies, utilizing both univariate and multivariate analyses to evaluate the effect of the oral microbiome on the survival of patients across different types of cancers. Moreover, a stratified analysis of the microbiome’s effect on survival in the Fusobacterium and P. gingivalis subgroups was also conducted. However, our meta-analysis does have several limitations. First, the inclusion of retrospective studies may have affected the results due to specific biases. Additionally, the evaluation criteria among the studies varied due to differences in the assessment methods, types of microbiomes, and samples. Lastly, even though we performed subgroup analysis, there were a limited number of studies included, so we did not present separate analyses for primary sites such as colorectal cancer or esophageal SCC. Besides, it is important to note that the oral microbiome also depends on factors such as dietary structure, living environment, and geographic location of the studied population.

## Conclusion

Drawing upon our findings, we have established that the oral microbiome represents a valuable prognostic factor for those afflicted with cancer. Specifically, patients with diminished diversity within their oral microbiome, or those exhibiting high levels of/positive Fusobacterium, or positive P. gingivalis within their cancerous tissue are at significantly higher risk of experiencing poor OS and DSS. Consequently, we recommend that great emphasis be placed on the effect of the oral microbiome in relation to cancer patient survival.

### Supplementary Information

**Additional file 1.** Search strategy.

## Data Availability

Data were extracted from published sources.
