# Supplementary material for: Prognostic impact of oral microbiome on survival of malignancies: a systematic review and meta-analysis
Source: Syst Rev. 2024 Jan 25;13:41. doi: 10.1186/s13643-023-02419-7 (PMC10809532; doi:10.1186/s13643-023-02419-7)
Supplement: Supplementary file 1 — Additional file 1. Search strategy. [file 13643_2023_2419_MOESM1_ESM.docx]

**Search strategy：**

The search terms were conducted as follows: “Oral Cavity,” “Cavity, Oral,” “Cavitas Oris,” “Vestibule of the Mouth,” “Vestibule Oris,” “Oral Cavity Proper,” “Mouth Cavity Proper,” “Cavitas oris propria,” “Oral and Maxillofacial Pathology,” “Oral Pathology,” “Pathology, Maxillofacial,” “Maxillofacial Pathology,” “Microbiotas,” “Microbial Community,” “Community, Microbial,” “Microbial Communities,” “Microbial Community Composition,” “Microbial Community Composition,” “Community Composition, Microbial,” “Composition, Microbial Community,” “Microbial Community Compositions” “Microbial Community Structure,” “Community Structure, Microbial,” “Microbial Community Structures,” “Human Microbiome,” “Human Microbiomes,” “Microbiome, Human,” “Microbiome,” “Microbiomes,” “neoplasia,” “neoplasias,” “neoplasm,” “tumors,” “tumor,” “cancer,” “cancers,” “malignancy,” “malignancies,” “malignant neoplasms,” “malignant Neoplasm,” “neoplasm malignant,” “neoplasms malignant,” “benign neoplasms,” “neoplasms, benign,” “benign neoplasm,” “Neoplasm, Benign,”
